# The efficacy of LIFESTYLE behavioral intervention in improving healthy eating and reducing alcohol consumption in patients with severe mental disorder: one year of follow-up

**DOI:** 10.1192/j.eurpsy.2025.2168

**Published:** 2025-08-26

**Authors:** B. Della Rocca, P. Catapano, C. Toni, M. Di Vincenzo, E. Mancuso, F. Lucci, G. Sampogna, M. Luciano, A. Fiorillo

**Affiliations:** 1 Università della Campania “Luigi Vanvitelli”, Napoli, Italy

## Abstract

**Introduction:**

Patients with severe mental disorders (SMD) often report unhealthy dietary patterns, including low intake of fruits and fiber, high consumption of junk food, and alcohol misuse, leading to poor nutritional status and increased oxidative stress, which negatively impacts physical and mental health. Psychoeducational interventions focusing on dietary habits and alcohol consumption have shown promising results, but long-term data is currently scarce.

**Objectives:**

The main objective was to evaluate the LIFESTYLE intervention’s one-year effectiveness in helping individuals with SMD improve their eating habits. Secondary objectives included evaluating the impact of psychiatric symptoms on lifestyle behaviors and the reduction of alcohol consumption.

**Methods:**

The study included 401 patients with SMD from 7 university centres in Italy. Participants were randomized to either the control group, which received general health education, or the experimental group, which received a 5-month psychoeducational intervention. The intervention featured group sessions focused on diet, physical activity, and behavior modification. Univariate analysis was performed to investigate the correlation between psychiatric symptoms and changes in lifestyle behaviours, such as eating habits, physical activity, and alcohol consumption.

**Results:**

Univariate analysis showed significant improvements in lifestyle behaviors among the experimental group. There was an increase in fish consumption (OR: 1.67, 95% CI: 1.45-1.97; p < 0.05), fresh fruit intake (OR: 1.36, 95% CI: 0.80-2.31; p < 0.05), and vegetable consumption (OR: 1.91, 95% CI: 1.56-1.96; p < 0.05). Moreover, there was a reduction in junk food consumption (OR: 0.814, 95% CI: 0.53-1.25; p < 0.05) and daily alcohol intake (OR: 0.70, 95% CI: 0.42-1.15; p < 0.05).

**Conclusions:**

The results of this study support the efficacy of structured lifestyle intervention for enhancing physical activity and eating behaviors in patients with severe mental disorders. They also support the translation of similar interventions into clinical practice and illustrate the necessity of physical activity and dietary advice in patients with SMD as part of their treatment schedule. Psychoeducational interventions can greatly improve the long-term health outcomes for those with SMD.

**Disclosure of Interest:**

None Declared

